# Diseases of the Lungs

**Published:** 1904-05-14

**Authors:** 


### Progress in Medicine and Surgery.

### DISEASES OF THE LUNGS.

(*Concluded from page* 102.)

**Bronchiectasis**, another common but interesting condition, has been recently reviewed by Habershon.[7] Setting aside the rare cases of acute bronchiectasis in children, the chronic cases are due to congenital weakness in the tubes, the various interstitial and parenchymatous inflammations of the lungs, obstruction from foreign bodies in the bronchi, compression by tumours, etc., from without, stenosis from ulceration, and the obstruction caused by adenoids—this latter being by no means an uncommon cause. Emphysema he regards rather as an effect of bronchiectasis. The various physical or inducing causes he considers can only act on previously weakened tubes. The physical signs vary with the amount and position of the lesion and the condition of the rest of the lung. He lays some stress on the croaking râles, on Laennec's "veiled puff," in which inspiration begins at a low pitch and ends in a sudden puffing sound, and "post-tussive suction," a sound due to the inrush of air into a previously collapsed cavity ; the last two sounds, however, are also heard in phthisical cavitation. The most characteristic symptoms are the paroxysmal cough, the profuse expectoration, the hæmoptysis, and the clubbed fingers. Pain is rare, and cyanosis due to concurrent disease. The diagnosis from tuberculous disease is by the variable nature of the physical signs, the nature of the cough, the absence of tubercle bacilli, and the general well-being of the patient. The discharge of a localised empyema through the lung is mainly distinguished by the history, and gangrene by its severe symptoms. Pharyngeal abscess must be excluded. In dealing with the complications he calls attention to cerebral abscess, which may be multiple, and osteo-arthropathy, in which the bone ends undergo painless enlargement. The main dangers lie in the septic complications and hæmoptysis. The treatment is (1) hygienic (warm, dry climate) ; (2) local. Among disinfectants creosote takes the first place. Oil of garlic in capsules may be tried or intratracheal injections of a drachm of 2 parts menthol, 2 parts guiacol, and 88 parts of olive oil. He also thinks intravenous injections of formalin do good ; in hæmoptysis if severe $\frac{1}{2}$-drachm doses of calcium chloride should be given every two or three hours.

**Pleurisy.**—It is now very generally recognised, as was first taught by the French pathologists, that many sero-fibrinous pleurisies are tuberculous in origin. As, however, the tubercle bacillus is scarcely ever found in the effusion the diagnosis is often very difficult. Bunting[8] has recently tested Widal and Ravant's observations on the cellular elements in simple and tuberculous pleurisies, and finds that though the cells vary with the age of the effusion their views are in the main correct. In septic pleurisies the white cells are mainly polynuclear, with few mononuclear and few endothelial scales. In simple and also in tuberculous pleurisies the white cells are nearly all mononuclear and there are many red blood-cells. The positive diagnosis of tubercle can, however, only be made by injecting the fluid into susceptible animals. In mechanical effusions the greater number of cells are endothelial, generally arranged in clumps or "placards." In the treatment of pleural effusion Lees[9] strongly recommends local application of ice-bags after aspiration. This may obviate the necessity for morphia. Wynter[10] points out the advantage of raising the foot of the bed, especially in apical cases, so that the fluid may gravitate to the apices where it hinders respiration less. In the treatment by aspiration, attention has been called by Perkins[11] to the danger of acute œdema of the lung which follows in some cases. It occurs mostly after the sudden withdrawal of a large amount of fluid, and is characterised by the expectoration of great quantities of albuminous fluid, which, however, is not chemically identical with the pleural effusion. The symptoms are severe, and go on frequently to cyanosis, cardiac failure, and death.

They may set in at once, but usually there is a little delay, occasionally amounting to two hours. Even an exploratory puncture is not without danger, and Oliver[12] has recently published three cases in which serious symptoms ensued, two of which were fatal. He considers the most likely explanation to be a reflex cardiac and inspiratory inhibition through the branches of the vagus which may be specially irritable owing to the antecedent disease. The treatment lies in the avoidance of the sitting posture, with stimulants and artificial respiration should failure occur. The treatment of empyema has been recently discussed at the New York Academy of Medicine.[13] Beck advocates treatment by free resection of ribs whereby solid masses of matter can be easily removed and the cavity properly drained. When the patient's condition is bad a preliminary operation may be done followed by resection. In tuberculous cases resection is the only chance. Cocaine is preferable to any general anæsthetic. Berg considers that in a small number of early cases incision for aspiration is sufficient; and Erdmann only incises empyemata in children under cocaine, and finds resection rarely necessary. Lloyd only resects the ribs in chronic cases, and considers very light anæsthesia necessary in order that the cough may help to expand the collapsed lung.

**Pneumothorax.**—The treatment of this condition by aspiration (in traumatic cases) has recently been advocated. Burghard[14] has recorded a case, occurring three days after extensive injury to the chest in a boy, which was very successfully treated by aspiration; while Godlee has performed the same operation by means of a trocar and canula. The latter method appears only applicable when the intrapleural pressure is greater than that of the atmosphere; a condition which has been proved to exist in some cases by West.[15] The mechanism of respiration in pneumothorax has been investigated by Edmunds.[16] From observations on a case which was operated upon, and from experiments on 15 animals (cats), he concludes that when the pleura is opened from without a peculiar form of respiration occurs which he terms "triphasic." A deep inspiration fills the sound lung, the glottis then closes, and an expiratory effort sends the air from the sound into the collapsed lung, distending it; then the glottis opens and air is expired from both lungs. When the recurrent laryngeal nerves are cut this action cannot take place, unless, as may happen in cats, the extrinsic muscles can close the larynx. He also noted that a pressure of a few inches of water in the pleura or the injection of 1 in 20 carbolic resulted in death from reflex inhibition, and that the anæsthetic employed acted much more powerfully when only one lung was acting.

**Rare Conditions.**—A case of primary carcinoma has been reported by Demarest[17] in which the diagnosis was made by the discovery of typical squamous cells in the sputum. The disease was unsuccessfully treated by *x*-rays, and was remarkable for continued hæmoptysis and the absence of pain and emaciation. Perkins[18] calls attention to the fact that syphilitic disease owing to its rarity is often considered tuberculous. Repeated examinations of the sputum if negative may suggest syphilitic disease, and he quotes two cases from recent literature in one of which the diagnosis was made from the presence of gummatous ulceration of the skin, and the other by absence of bacilli and the results of treatment. Both cases recovered. Loudon,[19] in treating of hydatid disease of the lungs, points out that death of the cyst is exceedingly rare; he also points out that the symptoms commonly ascribed to rupture of the cyst—fœtid expectoration, hectic fever—are really due to septic infection, and that an aseptic and ruptured cyst only gives rise to cough, sweat, expectoration, and hæmoptysis. Hæmoptysis and dyspnœa are frequent before the cyst has ruptured, but a positive diagnosis may be difficult except where bulging occurs, which is only in children. Cardiac and abdominal displacement by pressure never occurs in any marked degree.

[7] Clin. Jour., Dec. 30, 1903, p. 161; Jan. 6, 1904, p. 183. [8] Pract., Nov. 1903, p. 687. [9] Loc. Cit. [10] Lancet, Jan. 2, 1904, p. 13. [11] Pract., Nov. 1903, p. 692. [12] Lancet, Jan. 2, 1904, p. 26. [13] Med. Rec., Dec. 19, 1903, p. 994. [14] Lancet, Dec. 5, 1903, p. 1576. [15] Ibid, Dec. 19, 1903, p. 1742. [16] Brit. Med. Jour., Nov. 21, 1903, p. 1322. [17] Med. Rec., Jan. 16, 1904, p. 96. [18] Pract., Nov. 1903, p. 685. [19] Ibid., p. 695.

